# The Assessment of the Tendon of Insertion of the Iliopsoas Muscle in Dogs Using Diagnostic Ultrasound Has Good Intraobserver Consistency but Lacks Interobserver Consistency

**DOI:** 10.3390/ani16050711

**Published:** 2026-02-25

**Authors:** Krysta E. Bailey, Anke Langenbach, Brittany Jean Carr, Denis J. Marcellin-Little

**Affiliations:** 1 Veterinary Surgical Centers, Vienna, VA 22180, USA; baileyke10@gmail.com (K.E.B.); langenbach@lvssm.com (A.L.); 2 The Animal Hospital at Liberty Highway, Anderson, SC 29621, USA; bjc0510@gmail.com; 3 Department of Surgical and Radiological Sciences, University of California, Davis, Davis, CA 95616, USA

**Keywords:** iliopsoas, muscle injury, tendon of insertion, musculoskeletal ultrasound, veterinary sports medicine, dog

## Abstract

The iliopsoas muscle (IPM) is a flexor of the hip joint in dogs that is vulnerable to injury. Intraobserver and interobserver reliability of musculoskeletal ultrasound when evaluating the tendon of insertion of the IPM was studied. The IPM tendons of insertion of a randomly selected cohort were screened by one investigator and recorded. Musculoskeletal ultrasound recordings were separated into two groups of 20 dogs with presumptively normal IPM tendons of insertion and 32 dogs with one or two presumptively abnormal IPM tendons. Recordings were anonymized. The 104 tendons from these 52 dogs were independently reviewed twice by three observers. Abnormalities were detected in 6 of 40 presumptively normal IPM tendons (15%) and 59 of 64 presumptively abnormal IPM tendons (92%). Intraobserver repeatability of measurements of tendon dimensions was good (range, 0.24 to 0.76 mm), intraobserver reliability was good or excellent (range, 0.812 to 0.917), and intraobserver consistency was good (all coefficients of variation <20%). All measurements had poor interobserver consistency (intraclass correlation coefficients <0.500). Measurements of the tendon of insertion of the IPM have acceptable intraobserver repeatability, consistency, and reliability but have poor interobserver consistency, suggesting that efforts should be made to standardize the interpretation methods when using diagnostic ultrasound to evaluate problems affecting the tendon of insertion of the IPM in dogs.

## Simple Summary

The iliopsoas (IPM) is a muscle that is used by dogs to move their back leg forward. Injuries to the tendon at the end of the IPM are common in dogs, particularly active dogs such as those doing agility. In this study, we evaluated 104 IPM tendons from 52 dogs. Some tendons were normal, and other tendons were injured. We evaluated whether a veterinarian who used a portable ultrasound machine to evaluate the tendon of the IPM would find the same changes if they scanned a normal or abnormal tendon twice. We also evaluated differences between the findings of three veterinarians reading scans of these tendons. We found that when a veterinarian evaluated tendons twice, measurements were usually very close to each other, but when different veterinarians read scans of the same tendons, findings often varied among veterinarians. To make findings more consistent, efforts should be made to make sure that all veterinarians interpret scans of tendons of the IPM using the same technique.

## 1. Introduction

The iliopsoas muscle (IPM) is a muscle group composed of the psoas major and the iliacus muscles. The IPM originates on the ventral aspect of the lumbar vertebrae and ilium and inserts on the lesser trochanter of the femur. The IPM is a key flexor of the hip joint and stabilizer of the vertebral column. The IPM is active during daily activities, such as walking, running, and jumping [1,2]. Injuries to the IPM include thickening of the tendon, disruption of the muscle fibers, focal edema or hemorrhage, bursitis, and avulsion [3,4,5]. The frequency of IPM injuries has been reported to range from 7.8% to 32% in agility dogs [4,5,6]. The assessment of the IPM is therefore common and important. The IPM can be evaluated using palpation and can be imaged using computed tomography [7,8,9], magnetic resonance imaging [8,10,11], and musculoskeletal ultrasound (MSUS) [3,6,8,10,12]. The use of MSUS to evaluate the IPM in dogs appears to have increased, possibly because of lower cost relative to other imaging methods, portability, being noninvasive and free of ionizing radiation, and the ability to perform serial evaluations [13]. Visualization of the anatomic features of the IPM using high-resolution 8 to 10 MHz linear transducers has been reported in dogs [3,12]. Protocols for MSUS evaluation of the IPM have been established in equine and human medicine [8,14,15]. These protocols include the evaluation of the presence of pathology (muscle strain, changes in tissue echogenicity, bursitis). Information on the ultrasonographic appearance of the normal IPM and on the consistency of its assessment within or between observers is limited to a few publications [3,12]. Information describing intraobserver repeatability and reliability, and describing intraobserver and interobserver consistency, is lacking.

The objectives of the current study were to evaluate the repeatability, reliability, and consistency of measurements of the tendon of insertion of the IPM collected by several observers. We hypothesized that intraobserver repeatability would be acceptable, that intraobserver reliability would be good or excellent, that intraobserver consistency would be acceptable, and that interobserver consistency would be good or excellent. Here, acceptable repeatability was defined as having a standard deviation of repeated within-subject measurements of <1 mm or <1 severity grade. Acceptable intraobserver consistency was a coefficient of variation (CV) of repeated measurements of <20%, and good or excellent intraobserver reliability and interobserver consistency were intraclass correlation coefficients (ICC) ≥0.75, <0.9, and ≥0.9, respectively.

## 2. Materials and Methods

**Sample**—The study was approved by the Animal Care and Use Committee of Veterinary Surgical Centers, Vienna, VA (protocol #VSCR 20230209). The study followed the ARRIVE guidelines. Signed informed consent was obtained from owners before inclusion. The study used a sample of convenience of client-owned dogs recruited between January 2023 and August 2023. Dogs were eligible for inclusion if they were 1 year old or more, if their body weight was ≥10 kg, and if they were scheduled to undergo a procedure requiring sedation or anesthesia. To increase the likelihood that normal and abnormal IPM tendons would be evaluated, two groups of dogs were recruited: one with and one without an orthopedic problem affecting their pelvic limbs. A medical history, signalment, prior diagnostic imaging, and physical examination findings were collected for each patient. Patients were excluded from the study if factors prevented safe sedation or anesthesia. Dogs from the group without a known orthopedic problem affecting their pelvic limbs were excluded from the study if pelvic limb lameness was observed or a pain response to pelvic limb palpation was present.

**Ultrasound imaging**—Dogs were placed in dorsal recumbency with the thorax and abdominal region in a foam trough. The pelvic limbs were extended on the treatment table. The lateral ventral abdomen was clipped and aseptically prepared on the left and right sides. One hip joint was extended to 150° without internal or external rotation. Hip extension was confirmed using a plastic goniometer [16]. The pelvic limbs were stabilized with foam blocks, nonstick tape, and sandbags to maintain their position. Isopropyl alcohol (70%) was used as a coupling agent. The IPM was examined. The procedure was repeated for the other IPM.

A portable MSUS device with a 10 MHz linear probe (Butterfly iQ, Burlington, MA, USA) was used [17,18]. The MSUS device was connected to a high-resolution screen (iPad 10.2, Apple, Cupertino, CA, USA) with 810 pixels × 1080 pixels images. Images and videos were saved as PNG files. All MSUS images were acquired by observer A (K.B.), a third-year resident in veterinary sports medicine and rehabilitation with intermediate MSUS skills (approximately 1 year of experience). The left and right IPM were visualized using MSUS while the dogs were prepped for a procedure. The IPM was scanned at a depth of 6.0 cm (2.4 inches). Videos and still images were acquired from the IPM origin to insertion longitudinally and transversely. The third lumbar vertebra (L3) was located using palpation to identify the origin of the psoas major muscle. The muscle was visualized arising caudally from the transverse process. The psoas major muscle belly was visualized in a longitudinal orientation, ventral to the vertebral bodies of L2 to L7 [12]. The femoral nerve was observed between the psoas and iliacus muscles. When assessing tendon length, the transducer was moved caudally along the lumbar region and towards the femur to identify the femoral head and lesser trochanter. The transducer was perpendicular to the femoral neck to minimize anisotropy of the overlying joint capsule. The presence of bursitis was evaluated at the cranial aspect of the femoral neck. Some dogs required rotation and fanning of the probe medially to obtain a full view of the iliopsoas tendon insertion on the lesser trochanter. Imaging of the left and right IPM was repeated ≥5 min later.

The IPM scans were initially screened by observer A and were separated into two groups for anonymization and analysis: one group included dogs with IPM deemed normal upon initial screening, and one group included dogs with one presumptively abnormal tendon of insertion of the IPM or both. Videos and still images of each tendon were reviewed by the three observers for completeness and quality. Images that did not include the IPM body, musculotendinous junction (MTJ), tendon of insertion, and insertion site were excluded. Images with perceived distortion were excluded. One image from each IPM was selected for measurements. Additional images and/or videos from the same IPM were made available for review. Images and videos were anonymized and uploaded to a cloud-based system (https://cloud.butterflynetwork.com).

Three observers with varying MSUS experience independently reviewed the MSUS evaluations twice in the same order. Observer A’s profile is described above. Observer B (A.L.) was board-certified in veterinary surgery and veterinary sports medicine and rehabilitation with beginner MSUS skills (<1 year of experience). Observer C (B.J.C.) was board-certified in veterinary sports medicine and rehabilitation with advanced MSUS skills (>10 years of experience). All observers were allowed brief training on how to use the measuring system. During preliminary training, each observer read MSUS images of IPM from 5 dogs not enrolled in the study to practice measurements.

Observers evaluated nine parameters of the tendon of insertion of the IPM: the length of the tendon of insertion, the width of the tendon of insertion at the MTJ, at its midpoint (50%), and at the insertion site, muscle and tendon fiber disruption, heterogeneity, echogenicity, mineralization, and bursitis at the insertion site. Muscle or tendon fiber disruption was graded as absent, mild (<5% muscle involvement) with focal edema or hemorrhage, moderate disruption (>5% muscle involvement) with mild fiber tearing and increased edema/hemorrhage, or severe when significant fascial tearing with marked to complete muscle fiber disruption and marked hemorrhage/edema was observed [4,5]. Tissue heterogeneity was graded using a modified fiber alignment score [19]. Fiber alignment at the insertion site was normal when ≥75% fibers were parallel, mildly abnormal when 50 to 74% of fibers were parallel, moderately abnormal when 25 to 49% of fibers were parallel, and severe when <25% of fibers were parallel. Muscle echogenicity was graded as hypoechoic, normal, or hyperechoic [19,20]. Tissue mineralization was graded as absent, mild (signs of mineralization in ≤25% of tissues), or severe (>25%) [3,21]. Bursitis was graded as absent (normal), mild, or marked (causing impingement of the tendon of insertion) (Table 1) [22].

**Statistical analyses**—Statistical analyses were done using statistical software (SAS v. 9.4, Cary, NC, USA) for all dogs combined and for dogs separated into two groups, one group of 20 dogs where the IPM was initially screened as normal (40 tendons: 34 normal and 6 abnormal) and one group of 32 dogs where the IPM was initially screened as abnormal on one side or both (64 tendons: 5 normal and 59 abnormal). Each IPM was the observational unit for statistical analysis purposes and was considered an independent observation. Reliability was defined as the intraclass correlation coefficient and was calculated as follows: (intersubject variance)/(intersubject variance + within-subject variance), where the variances were obtained from an ANOVA [23]. Values <0.5 indicated poor reliability, between 0.5 and <0.75 indicated moderate reliability, between 0.75 and <0.9 indicated good reliability, and ≥0.9 indicated excellent reliability [24]. Reliability >0.75 was considered acceptable. Coefficients of variation (standard deviation of replicates/mean of replicates) were calculated from an ANOVA to determine measurement consistency. Methods with CV < 20% were interpreted as being highly consistent [25]. Repeatability was calculated as the variance (mean squared error) of the ANOVA model [26]. Within-case repeatability of <1 mm was considered acceptable. Thresholds for acceptable reliability, consistency, and repeatability were subjectively selected based on tendon size and on the amplitude of changes anticipated in abnormal IPM. The effects of abnormality on the measures were assessed with a mixed-model ANOVA that accounted for repeated measures for each IPM. The observer was included as a fixed effect. Normality of the model residuals was accepted when the Shapiro–Wilk W test was >0.95. Variables that were not normally distributed were rank-transformed prior to the ANOVA. Post hoc pairwise comparisons were done using Tukey’s HSD test. For all tests, values of *p* < 0.05 were considered statistically significant. For the one binary metric, a logistic regression was used to analyze the effect of abnormality on homo/heterogeneity. A chi-square value of < 0.05 was considered a statistically significant association.

## 3. Results

A total of 92 dogs were screened for enrollment. Forty dogs were excluded because a signed informed consent was not available (*n* = 8), sets of images were incomplete (6), images were distorted (2) or had embedded markings (7), or measurements were incomplete (17). Fifty-two dogs were included in the analyses; 27 were male, and 25 were female. Median body weight was 28 kg (range, 10 to 60 kg). Median age was 5 years (range, 1 to 13 years). For each dog, sonographic evaluation of the left and right IPM was performed in duplicate, for a total number of 208 sonographic evaluations of 104 IPM. Adverse events were not observed during the study.

**Figure 1:**
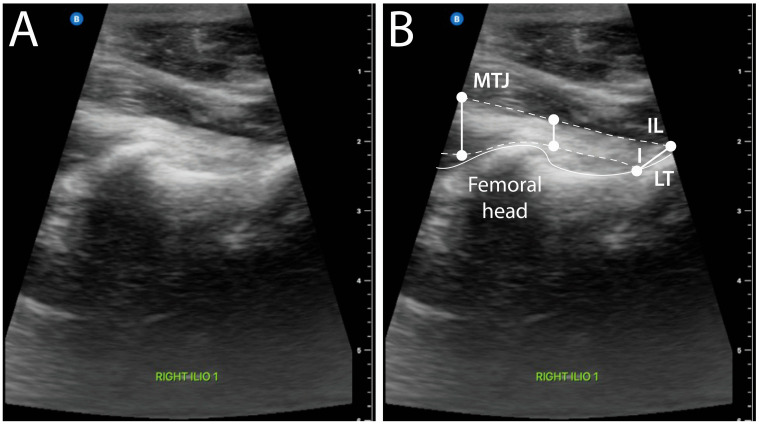
On this ultrasound image (**A**) and illustration (**B**) of a normal iliopsoas muscle from a 4-year-old American pit bull terrier weighing 20.4 kg, the muscle body can be seen from its musculotendinous junction (MTJ) to the lesser trochanter (LT), where the tendon inserts (I). Dashed lines follow the tendon outline. Tendon dimensions can be measured, including width at the musculotendinous junction and insertion site (IL), length, and width at the midpoint between the MTJ and I. For the dog shown, no fiber disruption was observed, tissue heterogeneity and echogenicity were considered normal, and mineralization and bursitis were absent. Musculotendinous junction width was 7.9 mm, tendon of insertion width was 6.6 mm, tendon length was 13.1 mm, and tendon width at the midpoint was 6.8 mm. A scale bar is shown on the right side of the images.

Twenty dogs had presumptively normal IPM at initial screening (Figure 1). For these dogs, 34 IPMs were normal, and an abnormality was detected by one observer or more in 6 of 40 tendons (15%). Median tendon length (range) was 13.5 mm (2.3 to 32.1 mm), median width was 6.8 mm (1.5 to 13.5 mm) at the MTJ, 4.6 mm (1.7 to 14.5 mm) at midpoint, and 12.0 mm (2.7 to 32.2 mm) at insertion.

For measurements of tendon length or width, the intraobserver repeatability ranged from 0.29 to 0.89 mm, the intraobserver consistency ranged from 5.0 to 20.6% (median, 11.4%), the intraobserver reliability ranged from 0.500 to 0.903, and the interobserver consistency ranged from 0.377 to 0.399 (Table 2). Seven of 12 measurements (58%) of tendon length or width had good reliability, and 1 (8%) had excellent reliability; all measurements had acceptable intraobserver consistency (<20%), and all measurements had poor interobserver consistency (<0.500).

**Figure 2:**
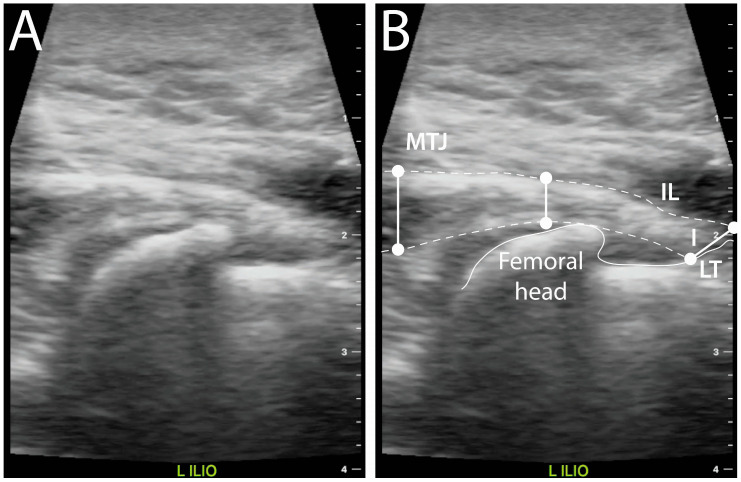
On this ultrasound image (**A**) and illustration (**B**) of an abnormal iliopsoas muscle from a 1-year-old mixed breed dog weighing 22.8 kg, the muscle body can be seen from its musculotendinous junction (MTJ) to the lesser trochanter (LT), where the tendon inserts (I). Dashed lines follow the tendon outline. Tendon dimensions can be measured, including width at the MTJ and insertion site (IL), length, and width at the midpoint between MTJ and I. For the dog shown, mild fiber disruption was observed; tissue heterogeneity was graded as mildly abnormal, with increased echogenicity, mild mineralization, and marked insertion site bursitis. Musculotendinous junction width was 10.3 mm, tendon of insertion width was 13.3 mm, tendon length was 18.0 mm, and tendon width at the midpoint was 8.0 mm. A scale bar is shown on the right side of the images.

Thirty-two dogs had unilateral or bilateral abnormal IPM at initial screening (Figure 2): 17 of these dogs had a cranial cruciate ligament injury, 13 had chronic hip dysplasia, 1 had medial patellar luxation, and 1 had a toe injury. For dogs in this group, an abnormality was detected by one observer or more in 59 of 64 IPM tendons of insertion (92%), and 5 tendons were normal. Median IPM tendon length (range) was 12.5 mm (4.6 to 29.9 mm), median width was 8.4 mm (3.6 to 20.0 mm) at the MTJ, 7.9 mm (2.8 to 15.5 mm) at the midpoint, and 10.9 mm (2.5 to 28.5 mm) at insertion. For measurements of tendon length or width, the intraobserver repeatability of measurements ranged from 0.24 to 0.76 mm, the intraobserver consistency ranged from 5.3 to 16.0% (median, 9.7%), the intraobserver reliability ranged from 0.812 to 0.917, and the interobserver consistency ranged from 0.159 to 0.396. Nine of 12 measurements (75%) of tendon length or width had good reliability, and 3 (25%) had excellent reliability; all measurements had acceptable intraobserver consistency (<20%), and all measurements had poor interobserver consistency.

For all IPM combined, an abnormality was detected by at least one observer in 65 of 104 IPM (63%). Nine of 12 measurements (75%) of tendon length or width had acceptable intraobserver consistency (<20%), 4 of 12 measurements (33%) of tendon length or width had good reliability, and none had excellent reliability; all measurements had poor interobserver consistency (Table 3).

## 4. Discussion

In the current study, ultrasound examinations of 104 IPM tendons of insertion from 52 dogs were performed by three independent observers. Nine parameters were evaluated: four parameters were measurements of tendon dimensions, and five parameters were structural tendon abnormalities. At 63%, the prevalence of abnormalities for the tendons examined in the study was high. That high prevalence was likely because multiple tendon parameters were evaluated. It is also possible that dogs with chronic orthopedic problems have a high prevalence of changes in the tendon of insertion of the IPM.

Because of its safety, practicality, and low cost of use relative to advanced imaging, diagnostic ultrasound is being used by some clinicians to evaluate the tendon of insertion of the IPM in dogs [3], particularly in sporting dogs [4,5,6], where muscle strains are common [27]. However, little is known about the internal and external consistency of measurements of tendon dimensions and the identification of pathologic features. The study presented here evaluated the internal (intraobserver) repeatability, consistency, and reliability, and the external (interobserver) consistency of measurements of four dimensional parameters and five pathologic findings for the tendon of insertion of the IPM in dogs. The IPM includes the psoas and iliacus muscles and a single tendon of insertion on the femur. The study focused on the tendon of insertion because most of the IPM pathology is concentrated in the distal portion of the IPM and because the anatomic location and dual nature of the muscle body complicate its assessment.

When measuring tendon dimensions, measurements had acceptable repeatability within all observers. We accepted the hypothesis that ultrasound measurements of the tendon dimensions for the IPM are internally repeatable. This acceptable repeatability is in agreement with findings of repeatability of ultrasound examination of the patella ligament thickness in humans, where repeated measurements differences ≤0.7 mm were reported in one study, and limits of agreement ranging from 0.4 to 1.5 mm were reported in another study [28,29]. In one study in humans, ultrasound measurements of rotator cuff muscle thickness were also highly repeatable, with repeated measurement differences < 0.1 mm [30]. All measurements of normal and abnormal tendon dimensions had acceptable internal consistency. We accepted the hypothesis that ultrasound measurements of IPM tendon dimensions are internally consistent. The internal reliability of measurements of IPM tendon dimensions was good or excellent for normal and for abnormal tendons. We accepted the hypothesis that the intraobserver reliability of ultrasound measurements of IPM tendon dimensions is good. These findings agree with the generally good reliability of measurements of human patellar tendon dimensions reported in one study [28]. Subjectively, the internal consistency and reliability of measurements in the current study was equal for observers with less experience compared to the highly experienced observer, suggesting that the training of all observers was appropriate.

The external (interobserver) consistency of measurements of tendon dimensions was poor for normal and for abnormal tendons, suggesting that subjective decisions impacted these measurements. We failed to accept the hypothesis that measurements of tendon dimensions are externally consistent. Linear measurements were reasonably repeatable and consistent overall, particularly for repeated measurements within an observer. Interobserver consistency was poor for measurements of tendon dimensions. Low interobserver consistency of tendon measurements may have been influenced by the relatively small size of the tendon, its potential curvature, and limited observer training. Repeatability was lowest for measurements of tendon width at the lesser trochanter, most likely due to incomplete imaging of the distal aspect of the tendon and the lesser trochanter. Subjectively, imaging of the IPM insertion site was made more challenging by the wide rectangular geometry of the probe used relative to the narrow geometry of linear probes used with traditional ultrasound machines. Scanning the insertion site of the IPM with the wide probe appeared particularly challenging in dogs with thicker muscles. Also, imaging of the IPM insertion site appeared challenging because tendon visualization on the tablet connected to the handheld MSUS device made visualizing the lesser trochanter more challenging than visualizing images on a larger, stationary screen connected to a traditional ultrasound machine. Suboptimal visualization may have led to incomplete imaging of the IPM insertion region. In one study of human patellar tendons, a lack of interobserver consistency was also reported for measurements near the site of origin of the patellar ligament [28]. The source of measurement differences among investigators warrants further investigation so that guidance for measurements is more specific and interobserver measurement consistency increases. Improving interobserver consistency will likely require the development of a standard scanning protocol for dogs and the implementation of specific operator training. In humans, standard protocols for ultrasound evaluation have been described for several muscles and tendons, including the rectus femoris, quadriceps tendon of insertion, patellar ligament, and diaphragm [31,32,33]. In one study in humans, the combination of a 2 h training workshop, consensus instructions, and case folders significantly increased the interobserver consistency of ultrasound measurements of polycystic ovaries [34].

When tendon abnormalities were evaluated, intraobserver measurements had good consistency, acceptable repeatability, and most often had good or excellent reliability. For abnormal findings within tendons, the consistency of measurements was lower than for linear measurements because the interpretation of fiber disruption, tissue heterogeneity, and echogenicity is inherently more subjective than linear measurements. Variability in the assessment of fiber disruption and echogenicity may have resulted from the lower image resolution and quality of the handheld ultrasound relative to traditional ultrasound, making the interpretation of fiber alignment and echogenicity more subjective for observers with varying experience levels. Measurement consistency would likely be higher if scans had been collected using linear probes connected to high-resolution ultrasound machines. The low interobserver consistency was not surprising, considering that interobserver variability is most often higher than intraobserver variability. Also, diagnostic ultrasound has been described as “one of the most operator dependent imaging techniques” [35]. In one study evaluating tendon abnormalities in the wrist and ankle of humans with rheumatoid arthritis, agreement (kappa [κ] values) for all tendons and pathology was only moderate (κ = 0.42), with fair level of agreement for the wrist region (κ ranging from 0.27 to 0.34) and moderate to good values for the ankle region (κ ranging from 0.47 to 0.62) [36]. In one study evaluating ultrasound measurements of muscle quantity and quality in humans, intraobserver reliability ranged from 0.82 to 1.00, while interobserver consistency ranged from 0.59 to 0.97 [37]. Similarly, in one study evaluating measurements of the iliofemoral ligament on the surface of the hip joint capsule, an inexperienced sonographer had an internal repeatability of 0.6 mm and an intraobserver error of 7.00%, an experienced sonographer had an internal repeatability of 0.4 mm, an intraobserver error of 4.75%, and an interobserver error of 10.91% [38]. The suboptimal consistency of measurements in the current study was likely amplified by the difference in experience level among observers.

This study has limitations. The tendons of insertion of the IPM were not palpated. Therefore, the accuracy of palpation to detect abnormalities of the tendon of insertion of the IPM could not be evaluated. While handheld ultrasound devices are convenient and affordable, handheld ultrasound devices likely produce images of lower quality than traditional cart-based ultrasound machines. In one study of hypotensive human patients, images were comparable [39]. In one trial, cardiac, lung, renal, aorta, and biliary ultrasound studies performed with handheld devices had lower sensitivity (78% vs. 93%) but equal specificity (92% vs. 92%) and diagnostic accuracy (89% vs. 93%) compared to traditional cart-based ultrasound machines [40]. In another study in humans, resolution, detail, and image quality of the handheld device were compared to traditional ultrasound [41]. The handheld device performed better than traditional ultrasound for spinal assessment related to local anesthesia, but underperformed for obstetric diagnostics.

Since measurements in the current study were collected under sedation or anesthesia, it is unclear whether the findings of the study can be extrapolated to measurements collected awake. In one study, where investigators compared measurements of muscle thickness collected using ultrasound and computed tomography in dogs, the authors stated that sedation or general anesthesia did not appear to influence measurements of muscle thickness [42]. Because the current study involved clinical patients and advanced imaging was not used, the accuracy of measurements and findings could not be determined. In the scientific literature, the accuracy of MSUS assessments varies widely. In one study evaluating the diagnostic performance of ultrasound to screen horses for dorsal tears of the deep digital flexor tendon, sensitivity was good (85%), specificity was moderate (60%), and accuracy was 70% [43]. In a study of the stifle joint in 13 dogs, sensitivity to detect cranial cruciate ligament injury was only 15%, but sensitivity to detect a meniscal injury was 82% [44]. In one study evaluating the accuracy of 2D and 3D ultrasound measurements of tumor volume in dogs with transitional cell carcinoma of the urinary bladder, 2D ultrasound measurements had a low accuracy [45].

This study indicates that measurements of the tendon of insertion are consistent within an ultrasonographer but are inconsistent among ultrasonographers. Future efforts aiming at increasing the consistency of MSUS measurements of the tendon of insertion of the IPM should investigate the impact of dog size and conformation, ultrasound device type and quality, ultrasound probe geometry, ultrasonographer experience, and scanning protocol on the accuracy and consistency of measurements.

## 5. Conclusions

We concluded from this study that measurements of the tendon of insertion of the IPM have acceptable intraobserver repeatability, reliability, and consistency but have poor interobserver consistency. Efforts should be made to standardize the evaluation methods when using diagnostic ultrasound to evaluate problems affecting the IPM in dogs.

## Figures and Tables

**Table 1:** Aspects of the iliopsoas tendon of insertion evaluated using musculoskeletal ultrasound.

Parameter	Grading Method (Grade)	Abnormality	Reference
IPM tendon length	Length measurement (mm)	-	-
IPM tendon width at the MTJ	Length measurement (mm)	-	-
IPM tendon width, midpoint	Length measurement (mm)	-	-
IPM tendon width, insertion site	Length measurement (mm)	-	-
Muscle and tendon fiber disruption	No disruption (grade 0)	None	[4,5]
	Mild disruption (grade 1)	Focal edema or hemorrhage	
	Moderate disruption (grade 2)	Mild fiber tearing Increased edema/hemorrhage	
	Severe disruption (grade 3)	Significant fascial tearing Marked or complete disruption	
Tendon heterogeneity (fiber alignment)	Normal (grade 0)	≥75% fibers parallel	[19]
	Mildly abnormal (grade 1)	50 to 74% of fibers parallel	
	Moderately abnormal (grade 2)	25 to 49% of fibers parallel	
	Severe (grade 3)	<25% of fibers were parallel	
Tendon echogenicity	Decreased (grade 0)	Hypoechoic tissues	[19,20]
	Normal (grade 1)	None	
	High (grade 2)	Hyperechoic tissues	
Tendon mineralization	Absent (grade 0)	None	[3,21]
	Mild (grade 1)	Mineralization in ≤ 25% of tissues	
	Severe (grade 2)	Mineralization in > 25% of tissues	
Bursitis at the insertion site	Absent (grade 0)	None	[22]
	Mild (grade 1)	Mild inflammation, no tendon impingement	
	Severe (grade 2)	Severe inflammation, tendon impingement	

Abbreviations: IPM, iliopsoas muscle; MTJ, musculotendinous junction.

**Table 2:** Consistency of ultrasound measurements of 39 normal and 65 abnormal tendons of insertion of the iliopsoas muscles from 52 dogs collected by 3 observers from a group of 20 dogs with presumptively normal iliopsoas muscles and a group of 32 dogs with unilateral or bilateral presumptively abnormal iliopsoas muscles.

Dogs	Parameter	Intraobserver Repeatability	Intraobserver Consistency * (%)			Intraobserver Reliability *			Interobserver Consistency *
20 dogs with 34 normal and 6 abnormal IPM	Length of tendon of insertion (mm)	0.82 mm	10.4	18.2	5.0	0.808	0.734	0.903	0.399
	Width of tendon at MTJ (mm)	0.29 mm	8.0	18.6	13.1	0.811	0.761	0.768	0.384
	Width of tendon at midpoint (mm)	0.36 mm	8.7	20.6	12.3	0.873	0.760	0.809	0.377
	Width of tendon at insertion (mm)	0.89 mm	7.1	12.0	10.8	0.716	0.500	0.602	0.387
	Fiber disruption (grade 0 to 3)	0.10 grade	340	-	-	0.831	-	-	−0.031
	Tissue heterogeneity (grade 0 to 3)	0.08 grade	-	-	-	-	-	-	−0.044
	Tissue echogenicity (grade 0 to 2)	0.51 grade	17.1	-	-	0.703	-	-	0.362
	Tissue calcification (grade 0 to 2)	-	-	-	-	-	-	-	-
	Bursitis (grade 0 to 2)	0.21 grade	1020	-	633	0.500	-	0.494	−0.080
32 dogs with 1 (n = 5) or 2 (n = 27) abnormal IPM: 5 normal and 59 abnormal IPM	Length of tendon of insertion (mm)	0.70 mm	8.9	12.8	5.3	0.851	0.872	0.907	0.396
	Width of tendon at MTJ (mm)	0.24 mm	9.8	11.7	12.0	0.812	0.887	0.844	0.206
	Width of tendon at midpoint (mm)	0.30 mm	5.9	16.0	12.8	0.917	0.895	0.861	0.300
	Width of tendon at insertion (mm)	0.76 mm	6.0	7.6	9.5	0.910	0.875	0.856	0.392
	Fiber disruption (grade 0 to 3)	0.63 grade	9.9	21.1	30.9	0.981	0.881	0.835	0.285
	Tissue heterogeneity (grade 0 to 3)	0.30 grade	-	-	18.2	-	-	0.886	0.192
	Tissue echogenicity (grade 0 to 2)	0.76 grade	58.3	94.7	30.3	0.966	0.862	0.821	0.274
	Tissue calcification (grade 0 to 2)	0.24 grade	-	450	406	-	0.744	0.639	0.233
	Bursitis (grade 0 to 2)	0.44 grade	-	106	93.2	-	0.934	0.896	0.159

Abbreviations: IPM, iliopsoas muscle; MTJ, musculotendinous junction; -, not available. * Intraobserver repeatability was the standard deviation of repeated within-subject measurements, intraobserver consistency was the coefficient of variation, intraobserver reliability was the intraobserver intraclass correlation coefficient (ICC) of each of 3 observers, and interobserver consistency was the interobserver ICC. Intraobserver consistency and reliability was calculated for each parameter and observer. Findings from observer A, B, and C are reported for intraobserver consistency and reliability.

**Table 3:** Consistency of ultrasound measurements of 104 normal (n = 39) or abnormal (n = 65) tendons of insertion of the iliopsoas muscles from 52 dogs collected by 3 observers.

Parameter	Intraobserver repeatability *	Intraobserver Consistency * (%)			Intraobserver Reliability *			Interobserver Consistency *
Length of tendon of insertion (mm)	0.27 mm	15.3	30.0	9.5	0.661	0.538	0.729	0.493
Width of tendon at MTJ (mm)	0.19 mm	12.0	35.0	14.9	0.724	0.483	0.780	0.355
Width of tendon at midpoint (mm)	0.23 mm	11.2	47.4	14.9	0.753	0.542	0.833	0.388
Width of tendon at insertion (mm)	0.26 mm	11.4	16.3	12.8	0.759	0.567	0.735	0.494
Fiber disruption (grade 0 to 3)	0.70 grade	84.2	92.3	82.0	0.746	0.580	0.705	0.288
Tissue heterogeneity (grade 0 to 3)	0.49 grade	86.5	88.5	82.2	0.622	0.446	0.593	0.095
Tissue echogenicity (grade 0 to 2)	0.65 grade	83.1	84.3	83.2	0.731	0.733	0.673	0.122
Tissue calcification (grade 0 to 2)	0.16 grade	383	494	611	0.699	0.863	0.483	0.244
Bursitis (grade 0 to 2)	0.36 grade	408	195	229	0.726	0.886	0.637	0.155

See Table 2 for legends.

## Data Availability

The raw data supporting the conclusions of this article will be made available by the authors without undue reservation.

## Abbreviations

The following abbreviations are used in this manuscript:

CV	Coefficient of variation
IPM	Iliopsoas muscle
MSUS	Musculoskeletal ultrasound
MTJ	Musculotendinous junction
